# Muscle-Restricted Nicotinamide Adenine Dinucleotide Phosphate Oxidase 4 Knockout Partially Corrects Muscle Contractility After Spinal Cord Injury in Mice

**DOI:** 10.1089/neur.2023.0089

**Published:** 2024-04-01

**Authors:** Carlos A. Toro, Rita De Gasperi, Abdurrahman Aslan, Nicholas Johnson, Mustafa M. Siddiq, Christine Chow, Wei Zhao, Lauren Harlow, Zachary Graham, Xin-Hua Liu, Junichi Sadoshima, Ravi Iyengar, Christopher P. Cardozo

**Affiliations:** ^1^ Spinal Cord Damage Research Center, James J Peters VA Medical Center, Departments of Icahn School of Medicine at Mount Sinai, Bronx, New York, USA.; ^2^ Medicine, Icahn School of Medicine at Mount Sinai, Bronx, New York, USA.; ^3^ Psychiatry, Icahn School of Medicine at Mount Sinai, Bronx, New York, USA.; ^4^ The Friedman Brain Institute, Icahn School of Medicine at Mount Sinai, Bronx, New York, USA.; ^5^ Pharmacological Science and Systems Biomedicine Institute, Icahn School of Medicine at Mount Sinai, Bronx, New York, USA.; ^6^ Healthspan, Resilience, and Performance, Florida Institute for Human and Machine Cognition, Pensacola, Florida, USA.; ^7^ Research Service, Birmingham VA Medical Center, Birmingham, Alabama, USA.; ^8^ Department of Cellular, Developmental, and Integrative Biology, University of Alabama-Birmingham, Birmingham, Alabama, USA.; ^9^ Department of Cell Biology and Molecular Medicine, Rutgers New Jersey Medical School, Newark, New Jersey, USA.; ^10^ Rehabilitation Medicine, Icahn School of Medicine at Mount Sinai, Bronx, New York, USA.

**Keywords:** muscle cKO, muscle contractility, Nox4, SCI mouse models, transection SCI

## Abstract

Spinal cord injury (SCI) results in severe atrophy of skeletal muscle in paralyzed regions, and a decrease in the force generated by muscle per unit of cross-sectional area. Oxidation of skeletal muscle ryanodine 1 receptors (RyR1) reduces contractile force as a result of reduced binding of calstabin 1 to RyR1. One cause of RyR1 oxidation is nicotinamide adenine dinucleotide phosphate (NADPH) oxidase 4 (Nox4). We have previously shown that, in rats, RyR1 was oxidized and bound less to calstabin 1 at 56 days after SCI by spinal cord transection. Here, we used a conditional knockout (KO) mouse model of Nox4 in skeletal muscle to investigate the role of Nox4 in reduced muscle specific force after SCI. Peak twitch force of extensor digitorum longus muscles in control mice after SCI was reduced by 42% compared with sham-operated controls, but was increased by ∼43% in SCI Nox4 conditional KO mice compared with SCI controls, although it remained less than that for sham-operated controls. Unlike what was previously observed in rats after SCI, the expression of Nox4 was not increased in gastrocnemius muscle, and binding of calstabin 1 to RyR1 was not reduced in this muscle. The results suggest that Nox4 is directly involved in reduction in muscle twitch force after SCI, although further studies are needed to understand the mechanistic basis for this linkage.

## Introduction

Spinal cord injury (SCI) results in sub-lesional paralysis and atrophy of skeletal muscles. In rodents, 40–60% of muscle mass is lost within the first few weeks after SCI.^1–3^ A similar although slower decrease has been reported in humans based on analysis of declines in muscle fiber cross-sectional area over the first 6 months after SCI.^4^ Muscle paralyzed by SCI is weaker and more easily fatigued. Rat soleus muscle was found to have a reduction in absolute force generation at 3 and 6 months after spinal cord transection associated with faster fatigue, a slow-to-fast transition of myosin heavy chain isoforms, and faster rates of contraction.^5^ Single fiber studies of rat tibialis anterior (TA) muscle at 2 and 4 weeks after a contusion SCI revealed reduced absolute force at 2 weeks post-SCI and reduced specific force (force produced per unit area) at 2 and 4 weeks after injury.^6^

The reasons for this reduced specific force generation after SCI are poorly understood. One possibility is impaired function of ryanodine receptor 1 (RyR1) in skeletal muscle. RyR1 is a large calcium-activated calcium channel present in the membrane of the endoplasmic/sarcoplasmic reticulum.^7–9^ In skeletal muscle, RyR1 is localized to the triad, a cellular structure formed by the junction of evaginations of the sarcoplasmic reticulum with T-tubules through formation of complexes of dihydropyridine receptors (L-type voltage-gated calcium channels localized to the cytoplasmic membrane/sarcolemma) and RyR1.^7–9^ Depolarization of the cytoplasmic membrane results in opening of RyR1 and release of calcium stored in the sarcoplasmic reticulum resulting in muscle contraction in a process referred to as excitation contraction coupling (ECC).

The open probability of RyR1 is regulated by several factors, including binding of calstabin 1, a small protein that stabilizes RyR1 in a closed state.^10^ Oxidation and nitrosylation of RyR1 decrease the binding of calstabin 1 to RyR1 and are associated with frequent bursts of calcium release through individual RyR1 channels (i.e., calcium sparks) that are thought to deplete sarcoplasmic reticulum calcium stores to reduce effectiveness of muscle contraction in response to neural activation of skeletal muscle. Administration of molecules that restore normal binding of calstabin 1 to RyR1 significantly improves muscle contractile function in animal models in which there is extensive oxidation and nitrosylation of skeletal muscle RyR1.^11–13^

The source of the reactive oxygen species (ROS) responsible for oxidative modifications of RyR1 may include nicotinamide adenine dinucleotide phosphate (NADPH)+ oxidases (Nox). Nox are widely expressed enzymes thought to be present in virtually all eukaryotic cells.^14–17^ They oxidize NADPH+ to generate ROS in response to physiological cues and participate in many physiological processes such as insulin action, regulation of vascular tone, and ECC.^15–17^ Six Nox isoforms are known.^14^ Nox4 is present in skeletal muscle where it has been found to form complexes with RyR1, and participates in oxidation in proportion to oxygen levels, thereby potentiating RyR1 opening and muscle contraction as oxygen becomes more abundant.^18^ Excessive expression of Nox4 mediates RyR1 oxidation and muscle weakness in mouse models of breast cancer bone metastases.^19^ Although Nox4 was initially believed to be constitutively active, more recent literature indicates that its activity is tightly regulated^16^ by tissue oxygen levels, cellular NADPH levels, and regulatory factors that include interactions with polymerase delta-interacting protein 2 (Poldip2).^20^

The possibility that muscle RyR1 becomes oxidized or nitrosylated following SCI has been addressed in a rat model of spinal cord transection. Our group has previously demonstrated that immunoprecipitated RyR1 isolated from rat gastrocnemius muscle at 56 days after SCI had elevated levels of oxidation and nitrosylation associated with almost complete loss of calstabin 1 binding.^21^ Expression of Nox4 was increased in gastrocnemius muscle at 56 days after SCI, and amounts of Nox4 co-immunoprecipitated with RyR1 were elevated after SCI,^21^ suggesting a mechanistic linkage between upregulation of Nox4 expression and RyR1 oxidation and nitrosylation. In the present study, we tested the hypothesis that upregulation of Nox4 is responsible for reduced specific force of skeletal muscle paralyzed by SCI. We conditionally knocked out Nox4 in cells of the myogenic lineage expressing the myogenic differentiation factor MyoD,^22,23^ using a mouse line in which the coding sequence for MyoD was replaced with that for Cre thereby driving Cre expression via the full-length murine MyoD promoter.^24^

## Methods

### Animals

Animals were housed in a temperature-controlled vivarium at ∼20°C with 12:12 h light–dark cycles, and were provided standard mouse chow and water *ad libitum*. All animal studies were conducted in conformance with the requirements of The Guide for Care and Use of Laboratory Animals and all other applicable regulations. Animal studies were reviewed and approved by the Institutional Animal Care and Use Committee at the James J. Peters VA Medical Center.

### Development of mice with conditional loss of function of Nox4 in skeletal muscle (cKO)

#### MyoD-Cre mice

A commercially available line in which the coding sequence for one allele of MyoD was replaced with an optimized Cre recombinase (iCre) (FVB.Cg-Myod1tm2.1[icre]Glh/J; Jackson Laboratories Stock No: 014140; MyoD-Cre)^24^ was used. MyoD is a member of the family of myogenenic regulatory factors that induce myogenic differentiation of progenitor cells.^25^ It is a transcription factor with a helix-loop-helix that when expressed together with other myogenic regulatory factors causes myogenic differentiation. Tissue expression of Cre has been evaluated in this MyoD-Cre mouse line by crossing it with a reporter gene line in which tissues express ß-galactosidase in the presence of Cre.^24^ Results confirmed expression of ß-galactosidase in limb skeletal muscles of embryos and in regenerating myofibers of skeletal muscles after inducing injury by intramuscular injection of cardiotoxin. No ß-galactosidase was observed in tissues other than skeletal muscle.^25^

#### Nox4 ^(f/f)^ mice

Mice in which exon 9 of the Nox4 gene is flanked by lox P sites (Nox4[f/f])^26^ were generously provided by Dr. Junichi Sadoshima (Rutgers New Jersey Medical School). Excision of exon 9 results in a new stop codon early in exon 10, thus causing translation of a truncated protein that lacks catalytic activity.^26^ This mouse model has been recently used to achieve cKO of Nox4 in heart,^26^ endothelial cells,^27^ and skeletal muscle.^28,29^

#### Generating MyoD-Cre/Nox4^(f/f)^ mice

Nox4^(f/f)^ mice were crossed with mice that were heterozygous for the MyoD-Cre allele. Progeny that carried one copy of MyoD-Cre and one copy of floxed Nox4 were crossed with Nox4^(f/f)^ mice. Resulting mice heterozygous for MyoD-Cre and homozygous for floxed Nox4 (cKO) were confirmed and used for subsequent breedings.

### Genotyping and confirmation of recombination

Genotyping was performed using genomic DNA isolated from ear snips obtained at the time of weaning. For detection of the Nox4 floxed allele, the following primers were used: primer 102, 5’ GCACTATGCCGAATTGCTCT; primer 103, 5’GAATGCACCGAGCACATTTG; and primer 105, 5’ GAGGCTATTCGGCTATGACT. The wild-type allele and the floxed allele were genotyped in separate polymerase chain reactions (PCR) combining primer 102 and 103 for the wild-type allele to generate a 900 bp band, and primers 102 and 105 for the floxed allele to generate a 1300 bp band. The reaction was performed with TopTaq polymerase and buffer (Qiagen) in the presence of 2 mM MgCl_2_ under the following conditions: 95°C for 3 min, 32 cycles at 94°C for 30 sec, 55°C for 30 sec, 72°C for 1 min 30 sec, and 72°C for 5 min. Genotyping for presence of the MyoD Cre allele was performed using the following primers: MyoD-Cre -wt-F, 5’ CGGCTACCCAAGGTGGAGAT; MyoD-Cre-Mut-F, 5’ GCGGATCCGAATTCGAAGTTCC; and Myo-Cre-R, 5’ TGGGTCTCCAAAGCGACTCC. The reaction was performed with TopTaq polymerase and buffer (Qiagen) in the presence of 2 mM MgCl_2_ under the following conditions: 95°C for 3 min, 35 cycles at 94°C for 30 sec, 60°C for 45 sec, 72°C for 45 sec, and 72°C for 10 min. The PCR products were 343 bp for wild type and 149 bp for the mutant allele; both bands were present for heterozygous mice.

To confirm recombination of the Nox4 gene at the genomic DNA level, DNA was isolated from biceps muscle using the DNeasy blood and tissue kit (Qiagen) according to the manufacturer's instructions. Here, genomic DNA (100 ng) was amplified by PCR using Taq polymerase and reagents from Qiagen and the following validated primers^29^: Fw: 5’ AACACTGTTGGACTCTTCAGACACA and Rv: 5’ CTCCTGATGCATCGGTAAAGTC. PCR amplification was performed for 35 cycles with the following conditions: 94°C for 30 sec, 62°C for 45 sec, and 72°C for 1 min. A band of ∼1600 bp generated by excision of exon 9 was detected in muscle from the cKO mice, but not in control Nox4^(f/f)^ muscle tissue, as previously reported.^29^

### Experimental design

Nox4 cKO mice and littermates carrying the floxed Nox4 but no Cre (Nox4^[f/f]^ genotype controls) were used at 4–6 months of age. Mice were randomly assigned to either spinal cord transection or sham transection surgeries. At 56 days after surgery, animals were deeply anesthetized by inhalation of isoflurane and the left extensor digitorum longus (EDL) was removed by careful dissection and then subjected to *ex-vivo* physiological testing. The remaining muscles were carefully removed by dissection, weighed, and snap frozen in liquid nitrogen.

### SCI surgeries and post-operative care

Animals were weighed and anesthetized by inhalation of isoflurane. Hair along the spine was removed with a clipper and skin was cleaned with 70% ethanol and beta-iodine solution. After exposing the spine through a midline incision centered at the T9-T10 intervertebral space, vertebral arches at T9-T10 were exposed by careful dissection and the T9 vertebral arch was carefully removed with sharp scissors. For animals assigned to the SCI group, a 0.1 mL of lidocaine solution was applied to the dura to anesthetize the area of injury, after which the spinal cord was severed with sharp microscissors. Completeness of transection was verified visually by retraction of ends of the severed cord. A small piece of surgical sponge was placed between the ends of the cord. Wounds were closed in layers using suture. Animals were administered carprofen and enrofloxacin pre-emptively after anesthesia but before the incision then for 3 days thereafter. Urine was expressed manually twice daily until spontaneous void of the bladder was observed. Thereafter, bladders were checked daily.

### Tissue harvest and *ex-vivo* physiology

Animals were weighed then anesthetized using inhaled isoflurane. Hindlimb muscles were excised after careful blunt dissection. Measurement of whole-muscle contractile and mechanical properties was performed using an Aurora Scientific *ex-vivo* physiology system for mice (Aurora, Ontario, Canada). Contractile properties of the EDL muscle were evaluated.

Briefly, a 4-0 silk suture was tied to the proximal and distal tendons of an intact right EDL, immediately distal to the aponeuroses. Following suture placement, muscles were then removed from the animal and immediately placed in a bath containing a Krebs mammalian Ringer solution at pH 7.4, supplemented with tubocurarine chloride (0.03 mM) and glucose (11 mM) for 10 min. The bath was maintained at 25°C and bubbled constantly with a mixture of O_2_ (95%) and CO_2_ (5%). The distal tendon of the muscle was then tied to a dual-mode servomotor/force transducer (Aurora Scientific, Aurora, Ontario, Canada) and the proximal tendon was tied to a fixed hook. Using wave pulses delivered from platinum electrodes connected to a high-power bi-phasic current stimulator (Aurora Scientific, Aurora, Ontario, Canada), each EDL was stimulated to contract. The 610A Dynamic Muscle Control v5.5 software (Aurora Scientific, Aurora, Ontario, Canada) was used to control pulse properties and servomotor activity, and to record data from the force transducer. Optimal voltage was determined by increasing the stimulation voltage of a single twitch pulse until force did not increase. This voltage was then doubled to ensure maximal stimulus. Optimal length (Lo) was then established by lengthening each EDL by 0.5 mm until force no longer increased following stepwise lengthening. Muscle was allowed to rest for 45 sec between twitch responses. Following determination of Lo, muscle was allowed to rest for 45 sec, and we collected twitch force.

After a 2-min rest, we then established the frequency–force relationship. Here, EDL muscles were stimulated at increasing frequencies (i.e. 10, 25, 40, 60, 80, 100, and 150 Hz). Stimulation was delivered for 300 ms, and muscles were left to rest for 1 min between successive stimuli. Maximum absolute isometric tetanic force (Po) was determined from the plateau of the frequency–force relationship. Muscles were then removed from the bath solution and weighed. All data collected were analyzed using the Dynamic Muscle Analysis v5.3 software (Aurora Scientific, Aurora, Ontario, Canada).

### Immunoprecipitation (IP) and Western blotting

IP of RyR1 was performed using buffers, antibodies, reagents, and procedures previously described.^11^ Briefly, gastrocnemius muscle was homogenized in 1 mL of 50 mM Tris HCl pH 7.4, 0.15 M NaCl, 0.5% Triton X-100 supplemented with Halt protease, and phosphatase inhibitors (ThermoFisher). The homogenate was centrifuged at 14,000 rpm for 20 min and the supernatant saved. Protein concentrations were determined using the BCA reagent (ThermoFisher). For IP, 0.5 mg protein was pre-cleared for 30 min at 4°C with 4% Agarose beads (ThermoFisher). To immunoprecipitate RyR1, 1.2 μg of anti-RyR1 monoclonal antibody (ThermoFisher MA 3-925) was added to the pre-cleared extracts and incubated 1h at 4°C. The samples were incubated with Protein A/G-agarose (Santa Cruz) for 1 h at 4°C. The beads were washed three times with the lysis buffer and heated at 95°C for 5 min. The immunoprecipitated proteins were separated by sodium dodecyl-sulfate polyacrylamide gel electrophoresis (SDS-PAGE) and transferred to a polyvinylidene difluoride (PVDF) membrane. After incubation in blocking buffer (Tris-buffered saline, 0.1% Tween-20 [TBST], 0.5 % non-fat dry milk) the membrane was incubated overnight with rabbit polyclonal anti-calstabin1 (PA 1-026, ThermoFisher) (1:1200 in blocking buffer). The membrane was washed with TBST and incubated for 1.5 h with HRP-conjugated anti rabbit IgG (1:10,000 in blocking buffer [Cytiva]) and the bands revelated with ECL Prime reagent [Cytiva].

To determine the amount of immunoprecipitated RyR1, the membrane was stripped and re-probed with the mouse monoclonal anti-RyR1 receptor antibody (MA 3-925, 1:2500). For Nox4 expression analysis, plantaris muscle tissue from Nox4^(f/f)^ and cKO was homogenized in 10 mM Na phosphate pH 7.4, 150 mM NaCl, 2 mM EDTA, 1% Triton X-100, 0.25% Na deoxycholate, 0.5% SDS supplemented with Halt protease, and phosphatase inhibitor cocktail (ThermoFisher) using zirconia beads and a FastPrep-24 tissue homogenizer (MP Biomedicals, Ohio, USA). Proteins (30 μg) were separated by SDS PAGE and blotted onto a PVDF membrane. The membrane was probed with a rabbit polyclonal anti-Nox4 (sc-30141, Santa Cruz) (1:500 dilution) following HRP-conjugated anti-rabbit IgG (1:10,000 in blocking buffer [Cytiva]). The bands were revelated with ECL Prime reagent (Cytiva). The membrane was then stained with Ponceau S Red staining solution (0.1% in 5% acetic acid, Sigma Aldrich) to visualize total protein load. Blots were imaged with ImageQuant 800 (Cytiva) and quantitated using the Image Quant TL software package (Cytiva).

### Enzyme-linked immunosorbent assay (ELISA) to detect protein carbonyl residues

Protein oxidation levels were quantified using an OxiSelect Protein Carbonyl ELISA Kit (Cell Biolabs, California, USA) according to the manufacturer's instructions. TA muscle was homogenized with ceramic spheres in the Fastprep-24 tissue homogenizer (MP Biomedicals, Ohio, USA) using 20 μL/mg RIPA buffer supplemented with a protease/phosphatase inhibitor cocktail (Cell Signaling Technology, Massachusetts, USA). The homogenized tissue was centrifuged at 14,000 rpm at 4°C for 10 min, and the supernatant was collected for analysis. Total protein concentration was calculated with the Bradford Assay (BioRad). The tissue lysates were treated with 1% DNase (Qiagen, Hilden, Germany) and diluted to a protein concentration of 10 μg/mL.

### Statistical analysis

Statistical calculations were performed using Graphpad Prism 9. Data are expressed as mean values ± standard error of the mean (SEM) and were analyzed by two-way mixed model analysis of variance (ANOVA) (KO x SCI) with a Sidak's test post-hoc to test for differences between pairs of means. Two-tailed *t* test was used to analyze Western blot expression data.

## Results

### Confirmation of Nox4 cKO

**FIG. 1.**
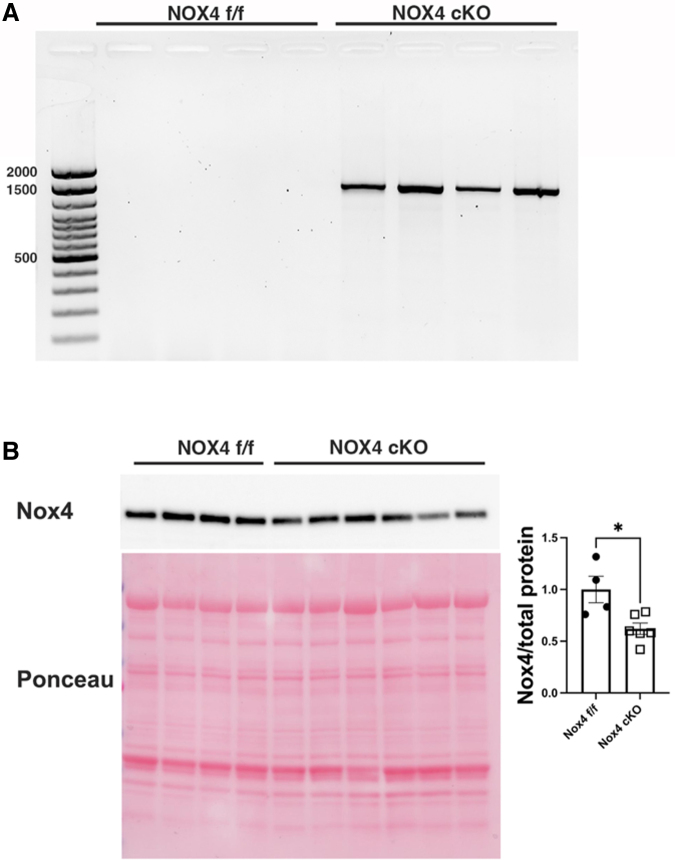
Confirmation of nicotinamide adenine dinucleotide phosphate oxidase 4(Nox4) genetic recombination and reduced expression levels in Nox4 cKO. **(A)** Representative gel image showing genetic recombination of Nox4 in the biceps of MyoD-Cre Nox4^(f/f)^ (cKO) animals. The ∼1600 bp band detected in cKO derives from the Nox4 gene that underwent excision of exon 9 by Cre recombinase. **(B)** Representative Western blot showing expression of Nox4 protein in Nox4^(f/f)^ and Nox4 cKO in plantaris muscle (upper panel) and Ponceau S Red stained membrane (lower panel) to detect total protein load. Nox4 levels were normalized to total protein load. Data were analyzed by two-tailed *t* test. *< 0.05.

Cre-induced recombination of Nox4 gene in muscle of MyoD-Cre Nox4^(f/f)^ was verified at the genomic DNA level by PCR designed to amplify the recombined Nox4 with exon 9 deletion. This analysis confirmed that recombination had occurred in muscle tissue from MyoD-Cre Nox4^(f/f)^ but not in Nox4^(f/f)^ genotype controls (Fig. 1 A). At the protein level, reduced expression of Nox4 was observed in the plantaris muscle of MyoD-Cre Nox4^(f/f)^ mice as compared with Nox4^(f/f)^ genotype control mice (Fig. 1 B).

### Body and muscle mass

**FIG. 2.**
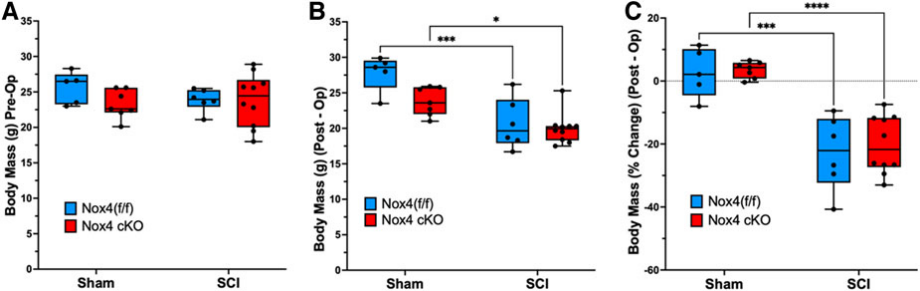
Changes in body mass after spinal cord injury (SCI). Body weights were determined at **(A)** the day of surgery (pre-operatively) and **(B)** the time of euthanasia (post-operatively). **(C)** Percent changes in body weight were compared with pre-operative body weight. Blue bars indicate nicotinamide adenine dinucleotide phosphate oxidase 4 (Nox4)^(f/f)^ Sham and SCI. Red bars indicate Nox4 cKO Sham and SCI. Data were analyzed by mixed-model two-way analysis of variance (ANOVA) with a Sidak's multiple comparisons test post-hoc. *p < 0.05; ***p < 0.001; ****p < 0.0001.

Animal body weights determined at both the day of surgery and the time of euthanasia (Fig. 2) were similar between SCI Nox4 cKO and SCI Nox4^(f/f)^ littermates. Body weights were reduced in the SCI groups regardless of genotype when compared with control, sham-operated mice at time of euthanasia (Fig. 2 B, C). Nox4 cKO and Nox4^(f/f)^ mice seemed to experience a similar degree of body weight loss after SCI (Fig. 2 C).

**FIG. 3.**
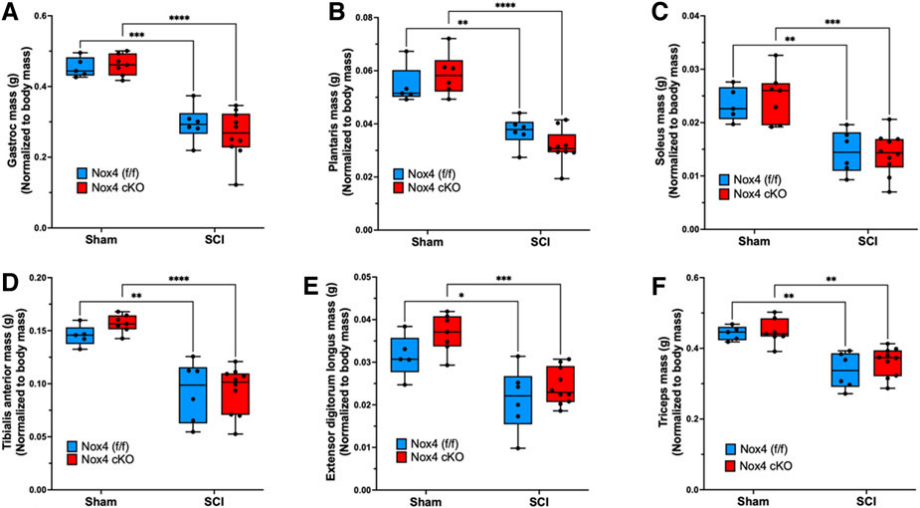
Weights of skeletal muscles are shown after normalization relative to pre-operative body weight. Normalized weights of the **(A)** gastrocnemius, **(B)** plantaris, **(C)** soleus, **(D)** tibialis anterior, **(E)** extensor digitorum longus, and **(F)** triceps muscles are shown. Blue bars indicate nicotinamide adenine dinucleotide phosphate oxidase 4 (Nox4)^(f/f)^ Sham and spinal cord injury (SCI). Red bars indicate Nox4 cKO Sham and SCI. Data were analyzed by mixed-model two-way analysis of variance (ANOVA) with a Sidak's multiple comparisons test post-hoc. *p < 0.05; **p < 0.01; ***p < 0.001, ****p < 0.0001.

Muscle weights were determined at 56 days after SCI and normalized to pre-operative body weight. For gastrocnemius muscle, normalized weights were similar between sham-operated Nox4 cKO and Nox4^(f/f)^ littermate controls (Fig. 3 A). Normalized gastrocnemius weights were significantly reduced in SCI Nox4 cKO and SCI Nox4^(f/f)^ groups compared with sham-operated controls (Fig. 3 A). There was no difference in muscle weight between SCI Nox4 cKO and SCI Nox4^(f/f)^ littermate controls (Fig. 3 A). The same pattern was observed for other lower hindlimb muscles including TA, EDL, soleus, and plantaris (Fig. 3 B–E). Weights of triceps muscles, a weight-bearing forelimb muscle that was not paralyzed by the SCI, were also determined. Triceps weights were similar between sham Nox4 cKO and sham Nox4^(f/f)^ mice, and between SCI Nox4 cKO and SCI Nox4^(f/f)^ mice (Fig. 3 F). Triceps weights were significantly reduced in SCI Nox4 cKO and SCI Nox4^(f/f)^ mice compared with sham-operated controls (Fig. 3 F). In summary, Nox4 cKO did not alter muscle mass in sham-operated mice or alter muscle atrophy in SCI mice at the 56-day time point studied.

### *Ex-vivo* physiology studies

**FIG. 4.**
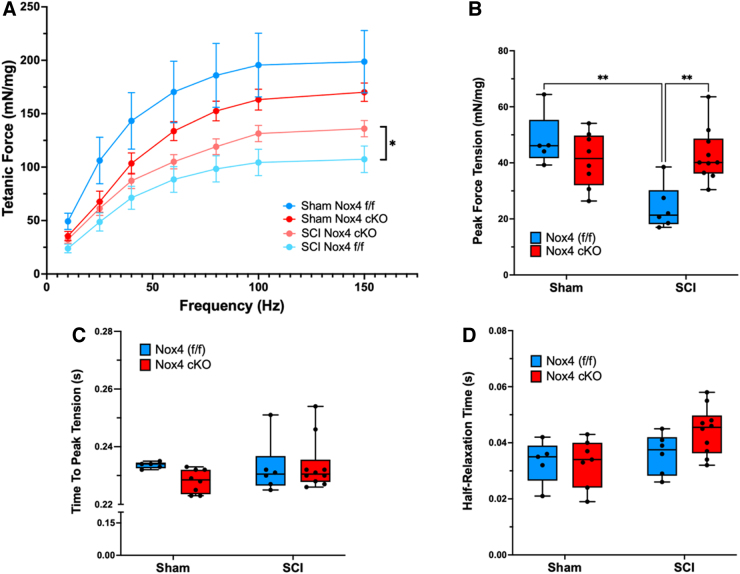
*Ex-vivo* physiological properties of extensor digitorum longus (EDL) muscles are shown. **(A)** Tetanic force-frequency was analyzed, **(B)** peak tetanic tension was measured at 150 Hz, **(C)** time to peak tension was determined, and **(D)** half relaxation time was assessed. Blue bars indicate nicotinamide adenine dinucleotide phosphate oxidase 4 (Nox4)^(f/f)^ Sham and spinal cord injury (SCI). Red bars indicate Nox4 cKO Sham and SCI. Data were analyzed by mixed-model two-way analysis of variance (ANOVA) with a Sidak's multiple comparisons test post-hoc. *p < 0.05; **p < 0.01.

To determine whether the Nox4 cKO improved muscle force generating capacity after SCI, EDL muscles were used for *ex-vivo* physiological studies. Analysis of force-frequency curves revealed a significant main effect for genotype in the SCI groups (*p* < 0.0001). Tetanic force generated by SCI mice with the Nox4 cKO was significantly increased as compared with SCI genotype controls (Nox4^[f/f]^) at 60, 80, 100, and 150 Hz (Fig. 4 A). Peak twitch tension tended to be lower in sham Nox4 cKO than in sham Nox4^(f/f)^ mice, although this difference did not reach the threshold for significance (Fig. 4 B). Peak twitch tension was significantly reduced for SCI Nox4^(f/f)^ compared with sham Nox4^(f/f)^ controls (Fig. 4 B). Peak twitch tension was significantly higher for SCI Nox4 cKO than for SCI Nox4^(f/f)^ and tended to be similar to that for sham-operated controls (Fig. 4 B). Time to peak twitch tension was not different between sham Nox4 cKO and sham Nox4^(f/f)^ groups and was not altered by SCI (Fig. 4 C). Half-relaxation time was also not different between any of the groups (Fig. 4 D). An unexpected pattern in the data was reduced muscle force generation in the sham Nox4 cKO mice compared with sham Nox4^(f/f)^ mice when EDL muscle was tested by force frequency studies or analysis of twitch contractions (Fig. 4 A, B); these differences did not reach the threshold for significance. In summary, the Nox4 cKO improved EDL force generation during twitch and did not appreciably alter time to peak tension or half-relaxation time.

### Binding of calstabin1 to RyR1

**FIG. 5.**
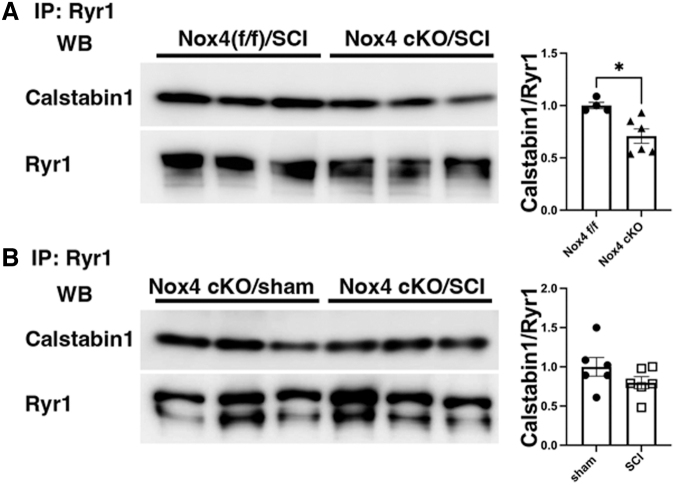
Binding and co-Immunoprecipitation of calstabin 1 with ryanodine 1 receptors (RyR1). **(A)** Representative Western blot showing co-immunoprecipitation of calstabin 1(FKBP12) and RyR1 in gastrocnemius muscle from nicotinamide adenine dinucleotide phosphate oxidase 4 (Nox4)^(f/f)^ and Nox4 cKO after spinal cord injujry (SCI) (*n* = 4 Nox4^(f/f)^, *n* = 6 for Nox4 cKO). **(B)** Representative Western blot showing co-immunoprecipitation of calstabin 1(FKBP12) and RyR1 in gastrocnemius muscle from Nox4 cKO sham treated as compared with Nox4 cKO after SCI (*n* = 6/group). Levels of calstabin 1 were normalized by levels of immunoprecipitated RyR1. Data were analyzed by two-tailed *t* test. (*p < 0.05).

Nox4 protein levels were not increased in Nox4^(f/f)^ mice at 56 days after SCI, in contrast to our prior observations in spinal cord transected rats.^21^ Co-immunoprecipitation experiments were performed to determine if binding of calstabin1 to RyR1 was altered after SCI in Nox4 cKO mice as compared with Nox4^(f/f)^. After SCI, there was decreased in binding of calstabin1 to RyR1 between Nox4^(f/f)^ and Nox cKO (Fig. 5 A). Calstabin 1 binding was not significantly different in SCI Nox4 cKO mice than in sham Nox4 cKO mice (Fig. 5 B).

### Protein carbonylation

**FIG. 6.**
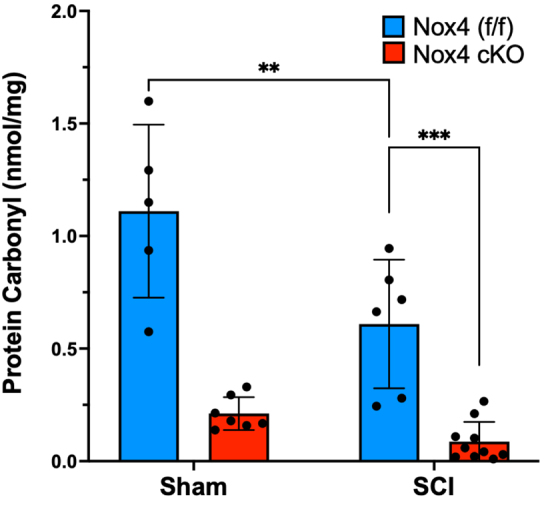
Nicotinamide adenine dinucleotide phosphate oxidase 4 (Nox4) increases oxidation of skeletal muscle proteins. Tibialis anterior (TA) muscle from controls (Nox4^[f/f]^) and Nox4 cKO were subjected to either sham surgeries or transection spinal cord injury (SCI). Protein carbonylation, a result of oxidation, was quantified by enzyme-linked immunosorbent assay (ELISA). Nox4^(f/f)^ animals produce significantly more protein carbonyls than Nox4 cKO animals, whether they received sham or SCI surgeries. Blue bars indicate Nox4^(f/f)^ Sham and SCI. Red bars indicate Nox4 cKO Sham and SCI. Data were analyzed by mixed-model two-way analysis of variance (ANOVA) with a Sidak's multiple comparisons test post-hoc. **p < 0.01; ***p < 0.001.

Protein carbonyls are produced by oxidation of amino acids such as lysine, arginine, proline, and threonine. Oxidation of these amino acids by ROS results in reactive ketones and aldehydes, which can be quantified by ELISA. Protein carbonyls were quantified in the TA muscle of SCI Nox4 cKO, SCI Nox4^(f/f)^, sham Nox4 cKO, and sham Nox4^(f/f)^ mice. Nox4^(f/f)^ TA had significantly more carbonylated protein than Nox4 cKO mice, regardless of whether the animals received SCI or not (*F* = 72.4, *p* < 0.0001) (Fig. 6). Sham Nox4^(f/f)^ mice had significantly more carbonylated proteins in TA muscles than SCI Nox4^(f/f)^ mice (*p* < 0.001). Sham Nox4 cKO mice appear to express more carbonylated proteins than SCI Nox4 cKO mice, but this difference did not reach the level of significance (*p* = 0.12).

## Discussion

This study used mice in which Cre was expressed under the control of the murine MyoD promoter such that exon 9 of the mouse Nox4 gene was excised at loxP sites in cells of the skeletal muscle lineage, thereby introducing a stop codon in exon 10 that prevented expression of a functional Nox4 protein. We compared outcomes between these mice and genotype control mice that carried the floxed Nox4 gene only. The major conclusion supported by the data is that loss of functional Nox4 in skeletal muscle improved muscle force generation and reduced protein oxidation in hindlimb muscles from mice at 56 days after spinal cord transection. The data support the hypothesized, mechanistic link among SCI, Nox4, protein oxidation, and reduced muscle contractility. The data do not support our hypothesis that in mice, Nox4 oxidizes RyR1 and thereby reduces binding of calstabin 1 to RyR1. Mechanisms by which reducing Nox4 expression in skeletal muscle increase muscle force generating capacity after SCI cannot be ascertained from the data here presented. Alternative mechanisms that have been identified in other mouse models of increased oxidative stress include decreased calcium responsiveness of contractile proteins, reduced sarcolemmal excitability, and alterations in homeostasis of cytosolic and sarcoplasmic reticulum calcium.^30^

We did not see complete knockdown of Nox4 in muscles of any of the mice tested. This may be because Nox4 is expressed in many different cell types. Nox4 has been reported to have important functions in neurons,^31^ vasculature,^32^ and skeletal muscle satellite cells.^33^ Although a detailed analysis of cell-type specific expression of Nox4 protein is not available, initial insights regarding expression of this gene in the cell types present in mouse skeletal muscle were obtained by searching data the Myoatlas single nucleus sequencing data set from the Milay Lab.^34^ A search of Myoatlas revealed low-level Nox4 expression in multiple cell types including myofibers, fibroadipogenic progenitors, endothelial cells, smooth muscle, and tenocytes. We believe that its expression in vasculature, smooth muscle, and nerves could explain why so much Nox4 protein is present in mice with conditional KO of Nox4 in MyoD-expressing cells. We considered the possibility that some truncated Nox4 protein could be translated. If this protein accumulates it would be expected to have a lower molecular weight. We did not detect bands at the expected molecular weight of this Nox4 variant.

Prior studies have shown that RyR1 oxidation reduces binding of calstabin 1, perturbing gating of RyR1.^11,12,19,35^ Consistent with predicted role of Nox4 in protein oxidation in muscle after SCI, we observed that the Nox4 KO reduced protein carbonylation in both sham and SCI mice. An unexpected finding was that muscle from sham Nox4 cKO mice appeared to contain more carbonylated proteins than that from SCI Nox4 cKO mice. This could reflect a role for Nox4 in exercise-induced adaptation of muscle, in sensing muscle oxygen levels, or other less well-defined roles of Nox4 in muscle physiology. It is also possible that catabolism of muscle proteins is more rapid after SCI as part of the muscle atrophy program. Consistent with this model, oxidized proteins are marked for degradation by the ubiquitin-proteasome pathway.^36^ There may also be a shift in muscle after SCI toward lower amounts of contractile proteins such that cytosolic, short-half-life proteins are more abundant, which could further reduce accumulation of oxidized proteins. Future studies are needed to sort out these mechanistic questions in a definitive manner.

Our results using Nox4^(f/f)^ mice differ from those we previously reported in Sprague Dawley rats.^21^ Why this difference exists is unclear. Possibilities include species or strain difference in expression levels of regulatory factors that govern Nox4 activity, or splice variants that determine how Nox4 is distributed or regulated. Nox4 is localized to many cellular compartments that include cytoplasmic membrane, nucleus, mitochondria, and endoplasmic reticulum^37^; it remains unclear whether there are species, tissue, or strain- dependent differences in Nox4 localization. Distribution of Nox4 in skeletal muscle has been reported,^28^ but the effects of SCI on Nox4 distribution are not yet known. Although we have found Nox4 bound to RyR1 in lysates of rat skeletal muscle,^21^ the cell type in which this colocalization occurs remains unknown although we have assumed it to be skeletal muscle fibers. Nox4 is regulated by multiple proteins,^16^ but the influence of species, strain, or muscle type on expression of the regulatory factors that govern Nox4 activity is unknown. Nox4 has also been found to be regulated by post-transcriptional modifications such as phosphorylation at Tyrosine 566 by tyrosine kinase FYN.^38^ Effects of SCI on expression or activity of FYN have not been examined. Further studies are needed to address these important questions.

The reduced force-generating capacity of skeletal muscles located in sub-lesional regions of the body is an important problem clinically because such impairments in muscle function reduce the potential functional benefit of the many exciting technologies now being developed to improve function of people with SCI. Some examples include spinal stimulation used alone or together with implanted brain electrode arrays to stimulate residual neural circuitry.^39^ An open question is whether increased physical activity and associated increased muscle workload can, with time, reverse the deleterious effects of SCI on protein oxidation and contractile force generation.

The current study was one of a growing number that have tested whether the skeletal muscle atrophy or reduced skeletal muscle force generating capacity observed after SCI can be blunted or reversed. In pre-clinical studies from our laboratories and others, a number of interventions have been tested including androgens,^40^ ActIIB receptor-Fc traps that inactivate myostatin,^41^ and ursolic acid.^42^ It is of note that to date, Nox4 cKO is the only intervention shown to increase any measure of specific force generation independently of neurological function. The observation that reduction of Nox4 expression increases muscle force production in completely paralyzed mice offers encouraging evidence that at least some of the defects in skeletal muscle after SCI can be prevented over the long term.

Further study is needed to understand the molecular basis for improved contractile capacity of muscle after SCI in Nox4 cKO mice and whether common mechanisms are operative across species. Such studies will need to examine well-known physiological determinants of skeletal muscle contractility that include calcium sensitivity of the contractile apparatus in studies with skinned fibers, evaluation of the level of oxidation of contractile proteins, and analysis of ryanodine-receptor and store-operated calcium entry into cytosol. Although our study did not find evidence of impaired RyR1-calstabin 1 binding, perturbations of this interaction should still be considered in future studies as a cause of reduced contractility that is potentially reversible given our findings in rats that SCI diminishes this interaction^21^ and the abundant evidence from the Marks laboratory that in mice, diminished calstabin 1 binding reduces skeletal muscle force production.^11,12,19,35^ It will also be important to understand how rehabilitation interventions such as functional electrical stimulation and spinal stimulation, which elicit neuromuscular activation, influence Nox4-related increased oxidative stress in muscle. Although on the one hand one expects that increased neuromuscular activation would reduce inappropriate activity of Nox4, it should also be considered that exercise induces Nox4 expression as part of the gene expression program that supports physiological adaptation to increased workload.^27^ Some insights can doubtless be obtained using electrical stimulation of peripheral nerves in rat and mouse models, as we have described.^43–45^

Our study has several important limitations. The first is that in the Nox4 cKO mouse model used, expression of functional Nox4 is blocked in all MyoD-expressing cells throughout the lifespan of the animals, which includes all cells of the myogenic lineage from proliferating satellite cells to mature myofibers. Although these mice had no obvious phenotype, we cannot exclude subtle developmental effects. Another limitation to consider is that conditional deletion of Nox4 could perturb normal physiology. Nox4 has been found to have roles in metabolic adaptations of skeletal muscle to physical activity,^27^ to act as an oxygen sensor that modulates muscle contractility in proportion to tissue oxygen levels,^18^ and to inhibit tissue repair in a mouse model of muscular dystrophy.^33^ Consistent with the previously reported role of Nox4 in increasing muscle contractility when tissue oxygen levels are high,^18^ we observed a small decrease in specific force- generating capacity of EDL muscle from MyoD-Cre/Nox4^(f/f)^ although this did not reach our threshold for significance. Further study is needed to fully understand how skeletal muscle-restricted inactivation of Nox4 impacts muscle physiology and responses to disease.

Our motivation for the studies reported herein was to improve the functional capacity of paralyzed muscles with the hope that the ever-increasing range of exciting rehabilitation therapies might achieve more rapid or more extensive gains in function. Examples of these therapies include spinal cell transplant-based therapies. This potential is more relevant to those injured >1 year earlier, in whom muscle atrophy is extensive and deconditioning is severe. Although our findings may have therapeutic potential through use of selective inhibitors of Nox4, or of drugs such as S107 (which normalizes calstabin 1 binding), the many roles of Nox4 in the physiology of the heart, nervous system, vasculature, and other systems suggests that a cautious approach will be needed. This is especially true for agents that downregulate or reduce the activity of Nox4. This is, nonetheless, a potentially exciting future direction for translational research.

## Conclusion

In conclusion, our study adds to the small but growing literature documenting physiological and pathogenic roles for Nox4 in skeletal and cardiac muscle, by demonstrating a critical role for Nox4 in increasing protein carbonylation and reducing *ex-vivo* contractility of paralyzed hindlimb muscles after spinal cord transection. Other functions of Nox4 in skeletal muscle include sensing oxygen tension,^18^ controlling metabolism,^27^ oxidizing RyR1 in mouse models of bone metastases of breast cancer,^19^ and regulating remodeling of dystrophic muscle.^33^ Critical roles have been demonstrated for Nox4 in the heart that include^26^ serving as the source of oxidative stress in heart failure.^26^ The availability of cKO and muscle-specific Cre expression systems should provide powerful tools for further elucidating the roles of Nox4 in normal physiology and disease.

## Abbreviations Used

ANOVA: analysis of variance
ECC: excitation contraction coupling
EDL: extensor digitorum longus
ELISA: enzyme-linked immunosorbent assay
KO: knockout
NADPH: nicotinamide adenine dinucleotide phosphate
Nox4: nicotinamide adenine dinucleotide phosphate oxidase 4
Poldip2: polymerase delta-interacting protein 2
PVDF: polyvinylidene difluoride
ROS: reactive oxygen species
RyR1: ryanodine 1 receptors
SCI: spinal cord injury
SDS-PAGE: sodium dodecyl-sulfate polyacrylamide gel electrophoresis
SEM: standard error of the mean
TA: tibialis anterior
